# The Immunology of Neuromyelitis Optica—Current Knowledge, Clinical Implications, Controversies and Future Perspectives

**DOI:** 10.3390/ijms17030273

**Published:** 2016-03-02

**Authors:** Michalina Jasiak-Zatonska, Alicja Kalinowska-Lyszczarz, Slawomir Michalak, Wojciech Kozubski

**Affiliations:** 1 Department of Neurology, Poznan University of Medical Sciences, 49 Przybyszewskiego St., 60-355 Poznan, Poland; wkozubski@ump.edu.pl; 2 Department of Neurochemistry and Neuropathology, Poznan University of Medical Sciences, 49 Przybyszewskiego St., 60-355 Poznan, Poland; akalinowskalyszczarz@ump.edu.pl (A.K.-L.); slamic@yahoo.com (S.M.); 3 Neuroimmunological Unit, Mossakowski Medical Research Centre, Polish Academy of Sciences, 5 Pawinskiego St., 02-106 Warsaw, Poland

**Keywords:** neuromyelitis optica (NMO), neuromyelitis optica spectrum disorder (NMOsd), immunopathogenesis, neuroimmunology, aquaporin-4 immunoglobulin G (AQP4-IgG), aquaporin-1 antibody (AQP1-Ab), myelin oligodendrocyte glycoprotein immunoglobulin G (MOG-IgG)

## Abstract

Neuromyelitis optica (NMO) is an autoimmune, demyelinating disorder of the central nervous system (CNS) with typical clinical manifestations of optic neuritis and acute transverse myelitis attacks. Previously believed to be a variant of multiple sclerosis (MS), it is now considered an independent disorder which needs to be differentiated from MS. The discovery of autoantibodies against aquaporin-4 (AQP4-IgGs) changed our understanding of NMO immunopathogenesis and revolutionized the diagnostic process. AQP4-IgG is currently regarded as a specific biomarker of NMO and NMO spectrum disorders (NMOsd) and a key factor in its pathogenesis. Nevertheless, AQP4-IgG seronegativity in 10%–25% of NMO patients suggests that there are several other factors involved in NMO immunopathogenesis, *i.e.*, autoantibodies against aquaporin-1 (AQP1-Abs) and antibodies against myelin oligodendrocyte glycoprotein (MOG-IgGs). This manuscript reviews current knowledge about NMO immunopathogenesis, pointing out the controversial issues and showing potential directions for future research. Further efforts should be made to broaden our knowledge of NMO immunology which could have important implications for clinical practice, including the use of potential novel biomarkers to facilitate an early and accurate diagnosis, and modern treatment strategies improving long-term outcome of NMO patients.

## 1. Introduction

### 1.1. The Definition of Neuromyelitis Optica

Neuromyelitis optica (NMO, previously called Devic’s disease) is an autoimmune, demyelinating disease of the central nervous system (CNS) manifesting with optic neuritis and acute transverse myelitis. Originally it was regarded as a monophasic syndrome that was a subtype of multiple sclerosis (MS) and consisted of bilateral simultaneous optic neuritis and acute transverse myelitis. Nowadays NMO is recognized as a typically relapsing, but sometimes monophasic disease with clinical, laboratory and neuroimaging characteristics that can differentiate it from MS. The most distinctive feature of NMO is the presence of the pathogenic antibody directed against aquaporin-4 water channel [1,2,3].

### 1.2. The History of NMO

The first account of a patient with visual loss and spinal cord disease was made by Antoine Portal in the 19th century. Other early descriptions included those by Pescetto, Durrant, Lockhard and Clarke [4]. In 1870 Allbutt recognized the association of visual loss and spinal cord disease. Ten years later Erb provided the first thorough description of NMO [1]. In 1894 a French neurologist, Eugene Devic, used the term “neuromyelitis optica” for the first time. Together with his student, Fernand Gault, he described a clinical syndrome characterized by optic neuritis and acute transverse myelitis [1,2,4]. Most initial reports described monophasic course of NMO [1]. Nevertheless, there were reports of patients with relapsing course of the disease (*i.e.*, by Beck in 1927 and McAlpine in 1938) [1]. In 2004 Lennon and Wingerchuk detected neuromyelitis optica immunoglobulin G (NMO-IgG), a specific marker antibody that differentiates NMO from MS [5]. A year later Lennon and colleagues discovered that NMO-IgG binds selectively to the aquaporin-4 (AQP4) water channel [6].

### 1.3. Diagnostic Criteria for NMO and NMO Spectrum Disorders (NMOsd)

In 2006 Wingerchuk and colleagues [3] formulated the revised diagnostic criteria for NMO which have been commonly used for several years. According to that scheme optic neuritis (ON), acute myelitis and at least two of three supportive criteria (continuous spinal cord magnetic resonance imaging (MRI) lesion encompassing over three vertebral segments, brain MRI not fulfilling diagnostic criteria for MS and aquaporin-4 immunoglobulin G (AQP4-IgG) seropositivity) should be present [3]. The above diagnostic scheme was believed to have sensitivity of 99% and specificity of 90% for NMO [3].

Subsequently, new discoveries in the field of NMO immunopathophysiology as well as some clinical and radiological features, pointed to the conclusion that a broader spectrum of clinical disease exists. The term “neuromyelitis optica spectrum disorders” (NMOsd) includes AQP4-IgG seropositive patients with NMO, but also other limited forms of the disease [2,7].

**Figure 1:**
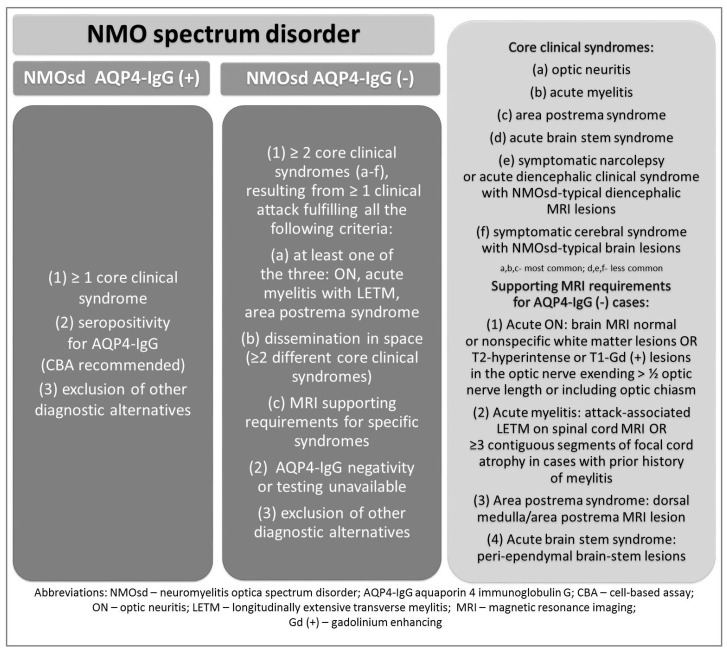
Diagnostic criteria for neuromyelitis spectrum according to International Panel for Neuromyelitis Optica (NMO) Diagnosis (2015) [8]. NMOsd: NMO spectrum disorders; AQP4-IgG: aquaporin-4 immunoglobulin G; MRI: magnetic resonance imaging.

In July 2015 the International Panel for NMO Diagnosis (IPND) published “International consensus diagnostic criteria for neuromyelitis spectrum disorders” [8]. According to the new criteria the general term of NMOsd should be used, which is further subdivided into NMOsd with or without AQP4-IgG on the basis of serologic testing, as shown in Figure 1 [8]. NMOsd with AQP4-IgG can be diagnosed when one of the six typical core syndromes is recognised (optic neuritis, acute myelitis, area postrema syndrome, acute brainstem syndrome, symptomatic narcolepsy or acute diencephalic clinical syndrome with NMOsd-typical diencephalic MRI lesions, symptomatic cerebral syndrome with NMOsd-typical brain lesions); and is associated with seropositivity for AQP4-IgG detected by the best available method when alternative diagnoses are excluded. The new diagnostic criteria for NMOsd without AQP4-IgG are stricter than those in the previous classification. Such a diagnosis can be made in a patient seronegative for AQP4-IgG when at least two core clinical features occur as a consequence of one or more clinical attacks, and all of the following conditions are met: (a) at least one core clinical feature must be NMO-typical (optic neuritis, acute myelitis with longitudinally extensive transverse myelitis (LETM) or area postrema syndrome); (b) clinically proven dissemination in space (two or more different core clinical syndromes) with additional MRI requirements specific for each clinical syndrome (see Figure 1) [8].

Importantly, the IPND recommends that an NMOsd diagnosis be made only when the patient has experienced at least one clinical attack [8]. Asymptomatic seropositivity for AQP4-IgG or asymptomatic MRI lesions characteristic for NMOsd are insufficient for the diagnosis [8]. Additionally, a single clinical attack of the disease is not diagnostic in AQP4-IgG seronegative patients [8]. Moreover, there are no clinical features that exclude NMOsd, but some of them might point to alternative diagnoses. Such red flags in NMOsd include the following: progressive clinical course with no connection between deterioration and disease relapse, atypical attack duration (<4 h or >4 weeks), partial transverse myelitis, oligoclonal bands in the cerebrospinal fluid (CSF), sarcoidosis, cancer, chronic infections (e.g., HIV, syphilis) and some MRI features (e.g., Dawson fingers, cortical lesions, peripheral spinal cord lesions or lesions of less than three vertebral segments) [8].

### 1.4. Clinical Features and Laboratory Findings

Neuromyelitis optica occurs more frequently in women than in men [2,7]. According to worldwide reports, female to male ratios range from 2:1 to 10:1. Moreover, up to 90% of relapsing NMO patients are women [7]. The median age of onset is 39 years, however, the disease may also occur in children and in the elderly [2]. The prevalence of NMO is about one to three per 100,000 [7]. The percentage of patients with NMO is low (1%–2%) in Caucasians, people from North America or Australia, and high (20%–48%) in people from the West Indies and Asia [2,9]. Neuromyelitis optica is a sporadic disease, but familial cases have also been reported (about 3% of patients) [7,9,10]. Due to the small number of familial cases, the lack of multigenerational pedigrees and the lack of distinctive characteristics of familial cases, the hypothesis that NMO is a complex genetic disease was made [11]. Some human leukocyte antigens (HLAs) are related to a higher risk of neuromyelitis optica, such as DRB1*0301 in white people, and people whose one parent is white and the other is black, and DPB1*0501 in Asian [2]. Furthermore, research showed the existence of antecedent factors associated with disease onset or clinical relapses, *i.e.*, postpartum period [7]. Moreover, the frequency of relapses might be higher in the last trimester of pregnancy and postpartum. Therefore, prophylactic treatment should be considered in these periods [9].

Neuromyelitis optica may be a monophasic or relapsing disease. According to the worldwide literature, 80%–90% of patients have a relapsing course. The most typical clinical features of NMO include ocular pain with impaired vision (optic neuritis) and acute transverse myelitis with paraplegia, sensory loss below the lesion and bladder dysfunction [2,7]. Nevertheless, other symptoms and signs may also occur: Lhermitte’s sign, paroxysmal tonic spasms or radicular pain can accompany acute myelitis, especially in patients with relapsing NMO [1,2,7]. Cervical myelitis extending into brainstem results in nausea, hiccups and even respiratory failure [2,7]. There are other uncommon clinical syndromes that might be present among NMO patients, including endocrinopathies, encephalopathy, coma, cerebral syndromes and the posterior reversible encephalopathy syndrome (PRES) [7]. After the first attack of the disease, 60% of patients experience another relapse within one year and 90% within three years [2,7]. Interestingly, AQP4-IgG seropositive patients with recurrent optic neuritis (ON) or the first episode of LETM are particularly at high risk of relapse [7]. Optic neuritis and LETM might occur simultaneously but usually they are separated by different periods of time [2,7]. NMO relapses are characterized by symptoms increasing over several days, and then slowly improving within weeks or months. Unfortunately, as opposed to MS, relapses are more frequent and more severe, followed by incomplete recovery, which leads to early, increasing disability [2,7]. Although monophasic patients experience more impairment from attacks than relapsing patients, their long-term outcome is better. After five years of disease duration over 50% of patients with relapsing course of the disease develop unilateral or bilateral blindness, or they need ambulatory help [2]. The proportion of patients with persistent monoplegia or paraplegia is also higher in the relapsing than the monophasic group of patients (52% and 31% respectively) [1]. Moreover, respiratory failure is also more frequent in the relapsing than in the monophasic patients (33% and 9% respectively) [1]. The prognosis of relapsing NMO is poor in comparison with MS [1]. The factors indicating a worse prognosis are the following: frequent relapses during the first two years of the disease, the high severity of the first attack and, interestingly, coexistence of systemic lupus erythematosus (SLE) or other non-organ-specific autoimmune disorders or the presence of autoantibodies [2]. The five-year survival is 90% in monophasic patients and 68% in relapsing patients. In the second group deaths are typically due to respiratory failure [1].

**Figure 2:**
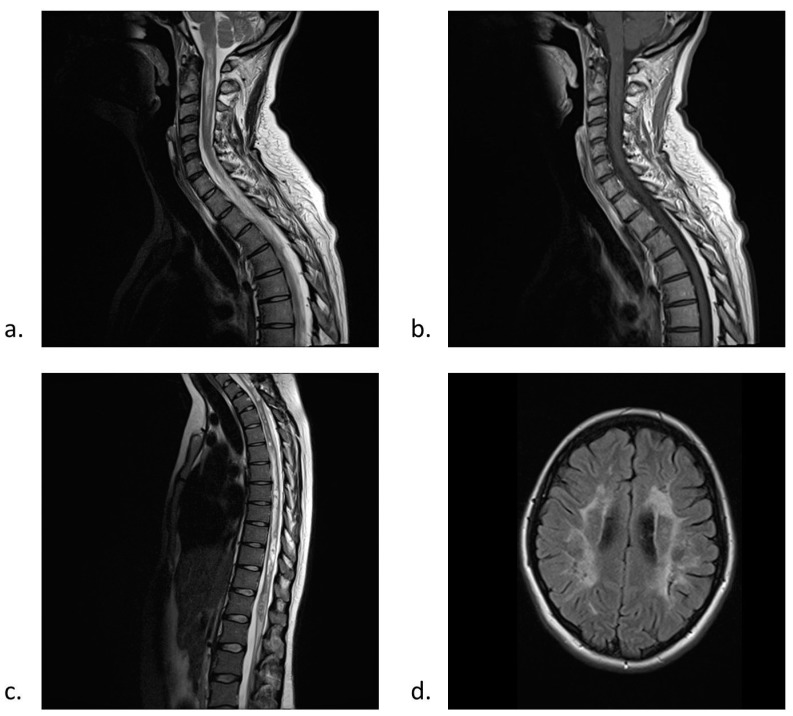
Neuroradiology of neuromyelitis optica spectrum disorder (NMOsd): (**a**,**b**) magnetic resonance imaging (MRI) visualisation of cervical spinal cord lesions in a 51-year old female with AQP4-IgG (+) NMOsd; (**a**) longitudinally extensive, central, extending to brainstem T2-hyperintense lesion; (**b**) corresponding T1-hypointensities, representing focal spinal cord atrophy; (**c**,**d**) thoracic spinal cord and brain MRI lesions in a 20-year old AQP4-IgG (+) female with NMOsd; (**c**) nearly complete central longitudinal thoracic spinal cord NMO involvement; (**d**) brain lesions on FLAIR (fluid-attenuated inversion recovery) images that do not fulfill multiple sclerosis diagnostic criteria. Images from the collection of Department of Neurology, Poznan University of Medical Sciences.

The initial brain MRI is typically normal, except for optic nerve gadolinium enhancement or brainstem lesions in some patients. Follow-up MRIs may reveal nonspecific brain lesions which are typically clinically silent and do not meet MS criteria [1,2,7] (see Figure 2d). Nevertheless, 10% of patients fulfilling the NMO diagnostic criteria might develop brain lesions in the follow-up MRIs that meet criteria for MS [2,7]. Moreover, it is reported that there are abnormalities in normal-appearing grey matter and normal or slight changes in normal-appearing white matter in magnetisation transfer (MTR) and diffusion tensor imaging (DTI) in NMO patients [2]. The above-mentioned data are the evidence of retrograde neuronal degeneration and selective or more severe destruction of grey matter marked by aquaporin-4 overexpression [2].

Spinal cord MRI has a great diagnostic value in NMO. It usually reveals longitudinal, continuous lesions extending across three or more vertebral segments [1,2,7] (see Figure 2a–c). These lesions are 98% sensitive and 83% specific for NMO [3]. Additionally, other typical findings are cord swelling and gadolinium enhancement [1]. Some patients develop focal spinal cord atrophy in follow-up MRIs [1] (see Figure 2b). It should be remembered that T2-weighted longitudinally extensive lesions may not develop fully in the first days after disease onset, however, they might contract or resolve over time [3].

Cerebrospinal fluid (CSF) examination may aid a differential diagnosis between NMO and MS. Significant pleocytosis (>50 cells/mm^3^) with a high proportion of neutrophils and high protein level (100 to 500 mg/dL) is common in NMO, unlike in MS [1,2,7]. Oligoclonal IgG bands are present in 85%–90% of patients with MS, but are uncommon in patients with NMO (15%–30%) [1,2,7].

Autoantibodies against aquaporin-4 (AQP4-IgGs, also called NMO-IgGs) are a specific NMO biomarker and play an essential role in the disease pathogenesis [2,5,6,9]. AQP4-IgGs are characterised by 73% sensitivity and 91% specificity for clinically defined NMO [2,5]. However, sensitivity depends on analytical methods used for the detection of AQP-4-IgG and is the highest for cell-based assays (CBA). AQP4-IgG can also be detected in other autoimmune disorders related to NMO such as Asian optic-spinal MS, recurrent ON, recurrent myelitis with LETM, ON or myelitis associated with certain organ-specific and non-organ specific autoimmune disorders [2]. Moreover, 10%–25% of NMO patients are seronegative for AQP4-IgG [2]. In sera of AQP4-IgG seronegative NMO patients two other autoantibodies were detected: autoantibodies against aquaporin-1 (AQP1-Abs) [11] and antibodies against myelin oligodendrocyte glycoprotein (MOG-IgGs) [12]. Moreover, some NMO patients are seropositive for both AQP4-IgG and AQP1-Ab [11].

In this manuscript we present a review of the current knowledge about NMO immunopathophysiology, pointing out the controversial issues and questions which still remain without unequivocal answer. Moreover, we show potential directions of future research into immunology and pathology of this devastating disease and their clinical, diagnostic and therapeutic implications.

## 2. Immunopathogenesis of NMO

### 2.1. Immunopathological Findings in NMO

**Figure 3:**
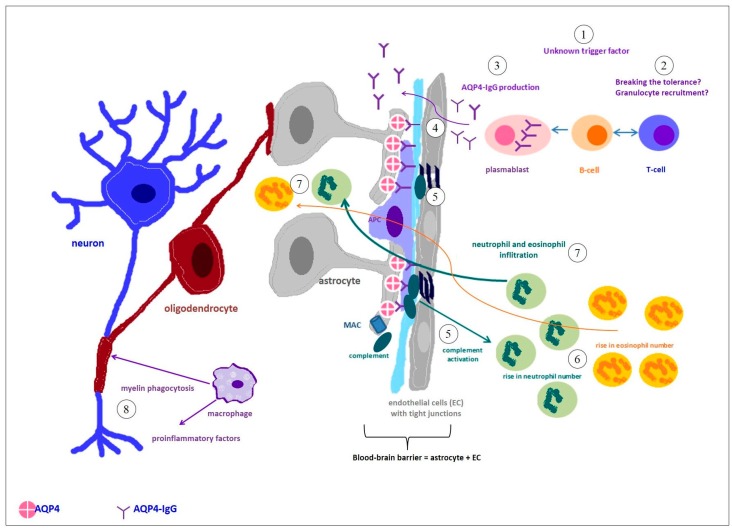
Immunopathogenesis of neuromyelitis optica: (**1**) primary immunizing event of unknown provenance; (**2**) T lymphocytes take part in breaking the tolerance and in recruitment of other leukocytes; (**3**) plasmablasts produce AQP4-IgGs, which enter the central nervous system (CNS) through (**4**) endothelial transcytosis or at areas of increased blood-brain barrier permeability and then bind selectively to aquaporin-4 (AQP4); (**5**) complement activation, leading to complement-dependent cytotoxicity and subsequent astrocyte death; (**6**) neutrophil and eosinophil rise in the periphery and their subsequent CNS infiltration (**7**) neutrophil and eosinophil infiltration (**8**) secondary demyelination caused by, among others, myelin phagocytosis, and bystander injury by pro-inflammatory factors. APC: antigen presenting cell; MAC: the membrane attack complex.

The immunopathology of neuromyelitis optica is different from that of multiple sclerosis [2,7]. Recent studies revealed a potential pathogenic role of AQP4-IgG, however, the primary immunizing event remains unknown [2]. AQP4-IgGs enter the CNS through endothelial transcytosis or at areas of increased blood-brain barrier (BBB) permeability and then bind selectively to aquaporin-4, a membrane protein forming the main water channel in the CNS. This interaction results in down-regulation of surface aquaporin-4 (AQP4) and perturbed water homoeostasis in the CNS. Moreover, it activates complement produced locally by astrocytes, which in turn leads to increased BBB permeability and massive infiltration of leukocytes, particularly eosinophils and neutrophils that can be found in the CSF during disease deterioration. Clonal expansion of B cells in the CNS is unusual in NMO, which explains the rare occurrence of oligoclonal IgG bands in the CSF. The combination of complement-mediated injury and cellular influx leads to the death of astrocytes, oligodendrocytes and neurons. Moreover, the complement membrane attack complex (MAC) causes changes in blood vessels located in NMO lesions, including their irregular thickening and hyalinization [2]. For a schematic NMO immunopathogenesis in a classical view (see Figure 3).

NMO lesions are marked by demyelination, which affects the grey and white matter, sometimes with necrosis and cavitation in the spinal cord and the optic nerves [2,7]. Demyelination characteristically extends across multiple spinal cord segments [2]. Unlike in MS, the inflammatory infiltration of active NMO lesions is composed of eosinophils and neutrophils, and the penetrating spinal vessels are thickened and hyalinized. According to immunopathologic examination, there is a characteristic vasculocentric rim and rosette pattern consisting of immunoglobulins and complement components in active NMO lesions [2,7]. The vasculocentric distribution of immunological complexes is mapped to the regions of AQP4 expression in the endfeet of astrocytes. NMO lesions are marked by decreased expression of AQP4 which distinguishes them from MS lesions. Moreover, the loss of AQP4 is accompanied by selective loss of astrocyte markers, e.g., glial fibrillary acidic protein (GFAP) [9].

Postmortem studies confirm that brain NMO lesions shown on MRI have the same immunohistochemical features as those in the spinal cord [2,7]. Such lesions are different from those seen in MS and acute disseminated encephalomyelitis (ADEM) [7].

Furthermore, the similarity of lesions found in Asian optic-spinal multiple sclerosis suggests that this disorder and NMO are a single clinical entity [2,7].

Some immunopathologic studies show a new type of NMO lesion localized in the spinal cord and medullary tegmentum, extending into the area postrema and marked by inflammation, edema and loss of aquaporin-4, but without demyelination or necrosis [2,7].

### 2.2. The Role of Several Cell Types in NMO Immunopathology

There are several types of immune cells important in NMO pathogenesis, including granulocytes, macrophages, natural killer (NK) cells, T lymphocytes and plasmablasts [9].

Several studies confirmed the essential role of granulocytes (neutrophils and eosinophils) in NMO [9,13]. Unlike in MS, the presence of both neutrophils and eosinophils is a very characteristic feature in NMO lesions [7,9,13]. It was reported that granulocytes and high concentrations of granulocyte colony-stimulating factor (GCSF) can be detected in patients’ CSF [9]. Neutrophil pathogenicity was studied in a mouse NMO model in which neutrophils escalated severity of NMO lesions, while in neutropenic mice the processes of neuroinflammation, myelin and AQP4 loss were significantly less intensified [14]. Moreover, immunostaining for neutrophil elastase (NE) showed many degranulated perivascular neutrophils [14]. Observations from this study point to the conclusion that complement activation by AQP4-IgG results in a marked rise in the amount of circulating neutrophils which enter the CNS and participate in early NMO lesions development through NE-dependent mechanism [14]. This was further confirmed by NMO lesion reduction caused by sivelestat, a neutrophil elastase inhibitor [14]. The precise role of eosinophils in NMO lesions development still remains unclear. Lucchinetti *et al.* [13] reported increased numbers of eosinophils in the spinal cord tissue of NMO patients [13]. Moreover, they confirmed the expression of CC-chemokine receptor-3 (CCR3), the major receptor for chemokine eotaxin, which is a potent eosinophil chemo-attractant, in NMO lesions [13]. Activated eosinophils release cytotoxic factors from granules, including eosinophil granule major basic protein (MBP), eosinophil-derived neurotoxin, eosinophil cationic protein and eosinophil peroxidase [13]. Whether eosinophil activation is a primary or a secondary event in NMO lesions development, remains unclear [13]. Complement activation results in the production of several chemotactic factors, such as component C5a, so the activation of eosinophils might be one of its consequences [13].

Another cell type present in NMO lesions are macrophages. Several studies revealed that macrophages might lead to axonal loss due to myelin phagocytosis and production of pro-inflammatory cytokines, glutamate, metalloproteases and free radicals in MS [9,15]. In NMO, macrophages could also scavenge the cell remains produced by astrocyte cytotoxicity and granulocyte infiltration [9,15].

It has been reported that T lymphocytes are involved in NMO immunopathogenesis [9,16]. This might be explained by the fact that AQP4-IgG belongs to IgG1 subclass whose activity depends on T cells. Moreover, Bradl *et al.* [16] found that AQP4-IgGs are able to cause NMO-like lesion development in Lewis rats under the condition that components of T-cell-mediated inflammation are present [16]. Several studies showed that T cells do not directly lead to development of NMO lesions [9], but they act in the periphery where they take part in breaking the tolerance, antibody production [9,16], and granulocyte recruitment into the CNS by inducing cytokine secretion from other immune cells [9]. The evidence supports the above-mentioned hypothesis, including the following: higher AQP4-IgG levels in serum than in the CSF, the small number of T lymphocytes in NMO lesions, NMO-like lesions in nude (lack of T cells) mice, the harmful effect of natalizumab (the antibody inhibiting CNS entry of T cells, but not neutrophils) in some NMO patients and the possibility of NMO lesions formation in patients after fingolimod treatment, which acts mainly by inhibiting T lymphocyte egress from peripheral lymph nodes [9].

According to several studies, interleukin-17-secreting T cells (Th17 cells), and interleukin-17A (IL-17A), which is produced by them, are involved in immunopathogenesis of autoimmune demyelinating diseases [17]. IL-17A is a cytokine inducing neutrophil attracting chemokine secretion from several cell types [17] and is produced by CD4+ T cells (Th17 cells) as well as CD8+ T cells, whose differentiation depends on TGF-β, IL-6 and IL-21 [18]. In turn, IL-23 is necessary for Th17 cell survival and function [18]. The relationship between Th17 cells, IL-17A and demyelinating lesion development has been particularly broadly described in MS [17]. Recent research supports a hypothesis about the essential role of Th17 cells also in NMO immunopathogenesis. Wang *et al.* [18] found that there are higher proportions of Th17 cells and IL-17-secreting CD8+ T cells in sera of NMO patients during relapse than in MS patients or the control group [18]. In addition, IL-17A and IL-23 levels are elevated in sera of NMO as well as MS patients [18]. In turn, IL-21 levels are increased in sera of NMO patients [18]. Higher numbers of Th17 cells, IL-17-secreting CD8+ T cells and IL-17A in patients with NMO in comparison with those with MS suggest that inflammation and demyelination is more severe in NMO than in MS [18]. Li *et al.* [19] showed that the numbers of memory Th17 cells, IL-17A and IL-23 are remarkably increased in sera of NMO and MS patients. Moreover, high levels of Th17 and memory Th17 cells are associated with the degree of disability measured by the Expanded Disability Status Scale (EDSS) and relapse frequency in NMO and MS patients. Additionally, high-dose intravenous methylprednisolone therapy (HIMP) causes a decrease in the number of Th17 cells and a suppression of their function [19].

It is known that NK cells can cause antibody-dependent cellular cytotoxicity (ADCC) [20]. NK cells account for 10%–20% of peripheral blood leukocytes and are the essential effector cells in cancer therapy [20]. Studies have shown that AQP4-IgG, together with NK cells, can lead to AQP4-transfected cell death in human astrocyte culture [20]. NK cells are not present in large numbers in NMO lesions, but their short lifetime after activation hinders evaluation of their possible involvement in NMO pathogenesis [20].

Research has shown that a B-cell subpopulation with the CD19^int^CD27^high^CD38^high^CD180^−^ phenotype, showing morphological and phenotypical characteristics of plasmablasts (PB), is involved in NMO immunopathogenesis. This B-cell subpopulation is significantly elevated in the blood of AQP4-IgG seropositive patients with NMO or NMOsd. Plasmablasts are responsible for AQP4-IgG production and they are a major source of these antibodies in the peripheral blood. The frequency of the CD19^int^CD27^high^CD38^high^CD180^−^ cells is correlated with the serum AQP4-IgG titers and increases further during a relapse [21]. It is reported that plasma cells might also be detected in the CSF of NMO patients and are a potential source of AQP4-IgG synthesized intrathecally [22]. The function of plasmablasts is connected with their exposition to interleukin 6 (IL-6). Several studies revealed that IL-6 is elevated in sera and CSF of NMO patients and enhances the survival of plasmablasts as well as promotes AQP4-IgG production [21]. The blockade of IL-6 receptor (IL-6R) signalling by anti-IL-6R antibody reduces the survival of plasmablasts *in vitro* [21]. This supports the hypothesis about an IL-6-dependent B-cell subpopulation role in NMO pathogenesis, and IL-6R blockade with tocilizumab might be a promising treatment for some NMO patients [21].

Recent research revealed new potential factors that might be involved in immunopathogenesis of NMO, including AQP1-Ab and MOG-IgG. However, further research is needed in this context [11,12].

## 3. Autoantibodies against Aquaporin-4 (AQP4-IgG, NMO-IgG)

### 3.1. AQP4-IgG as a Specific NMO Biomarker

NMO-IgG was first detected in sera of NMO patients in 2004 by Lennon and Wingerchuk. Subsequently, it turned out that NMO-IgG binds selectively to aquaporin-4 [2,6]. This discovery changed our understanding of neuromyelitis optica which turned out to be an autoimmune astrocytopathy and not primarily demyelinating disease [6]. AQP4-IgG is regarded as a specific NMO biomarker that allows to differentiate between this disease and other demyelinating disorders of the CNS [2].

### 3.2. AQP4—Function, Structure, Expression in the CNS and Other Organs

Aquaporin-4 functions mainly by maintaining water homeostasis [2] via regulation of extracellular space volume and interstitial fluid resorption [23]. Additionally, AQP4 has several other functions such as taking part in potassium buffering, CSF circulation, waste removal, neuroinflammation, osmosensation, cell migration as well as calcium signalling. Moreover, AQP4 is necessary for the normal function of the retina, the olfactory system and the inner ear [23].

Aquaporin-4 monomers are composed of six helical, transmembrane domains and two short helical segments located around an aqueous pore [9,24]. Aquaporin-4 monomers are expressed in astrocytes in two isoforms: M1 (with translation initation at Met-1) and M23 (with translation initation at Met-23). Both isoforms bind to form tetramers in the cell plasma membrane. The M23 subtype further aggregates to form supramolecular assembles named orthogonal arrays of particles (OAPs) or square arrays, whereas M1 exists as a tetramer and is unable to form large arrays alone [7,9,25,26]. The function of OAPs under physiological conditions is unknown [26]. However, in several CNS pathologies alterations in OAPs structure occurs [26]. Several studies revealed that AQP4 tetramers do not contain AQP4-IgG epitope and that AQP4-IgGs bind preferentially to OAPs [9,26,27]. Moreover, research suggests that OAPs might by essential for complement-dependent cytotoxicity due to multivalent complement component C1q binding to clustered AQP4-IgG [9,28].

In the CNS aquaporin-4 is expressed at high levels in the spinal cord, optic nerves, brainstem, hypothalamus and periventricular regions. It is also present in perivascular, periependymal and subpial regions, the area postrema and the supraoptic nucleus [7]. At the cellular level, AQP4 is expressed mainly in foot processes of astrocytes. It is also present in the so-called supportive cells in the sensory organs, such as Müller cells in the retina [9]. The immunohistochemical staining pattern shows that in the CNS AQP4-IgGs bind to the abluminal surface of microvessels, pia, subpia and Virchow-Robin sheaths [2,6]. CNS areas of the highest AQP4 expression tend to correspond with localization of brain lesions on MRI [2]. In 10% of cases unique brain NMO lesions with hypothalamic, corpus callosal, periventricular, or brainstem localization are observed [2].

Outside the CNS AQP4 is present in the distal collecting tubules in the renal medulla and in basolateral membranes of parietal epithelial cells in the gastric mucosa as well as in the airways, glands and skeletal muscles [2,9].

Interestingly, in NMO, pathological changes occur in the optic nerves, the spinal cord and sometimes in the brain, but no abnormalities are found in peripheral AQP4-expressing organs [9]. There are several proposed explanations for this fact. Firstly, in comparison with the CNS AQP4-expression is lower in the peripheral nervous system, also the role of AQP4-expressing cells is less important for tissue function in the periphery and there is higher expression of M1-AQP4 than M23-AQP4 [9]. Secondly, AQP4-expressing cells in the peripheral nervous system have increased resistance to complement-dependent cytotoxicity (CDC) due to lower expression of complement regulator proteins including CD46, CD55 and CD59 [9]. In addition the absence of some key inflammatory cascade components, such as microglia, could be the reason for lack of abnormalities in the peripheral organs [9]. Finally, normal renal function in NMO might indicate that aquaporin-4 contribution in water homoeostasis is more significant in the CNS than in the nephron [2].

### 3.3. The Role of AQP4-IgG in NMO Pathogenesis

#### 3.3.1. Evidence Supporting Pathogenicity of AQP4-IgG in NMO

A number of worldwide studies provide evidence for a potential role of AQP4-IgGs in NMO immunopathogenesis [2,7,9,16].

Clinical observations support the hypothesis that AQP4-IgGs lead to development of NMO lesions. Firstly, AQP4-IgGs are highly specific (85%–99%) for NMO and can be detected in sera of most patients (68%–91%) [2,7,9]. AQP4-IgG serum levels change with disease activity and treatment status [9,29,30]. Brain lesions visualized on MRI are located in regions of high AQP4 expression [7]. Finally, there is growing evidence that NMO patients may benefit from B-cell targeted therapy such as rituximab or plasma exchange [7].

Immunopathological studies showed that AQP4 immunoreactivity is localized in a perivascular rim and rosette pattern which matches the pattern of IgG and activated complement components deposition in NMO lesions. This suggests a role of humoral immunity in NMO pathogenesis [7,9,13]. Moreover, NMO lesions are marked by loss of AQP4 from the surface of astrocytes [7,16,31]. AQP4 is internalized and degraded, which results in AQP4 loss [31]. *In vitro* studies revealed that AQP4-IgGs lead to AQP4 modulation in cultured astrocytes and in non-neuronal cells expressing AQP4 transgenically [31,32]. Another key feature of NMO lesions is that inflammatory infiltration is connected with large amounts of perivascular granulocytes (neutrophils and eosinophils) and macrophages with relatively small amounts of T lymphocytes (CD3+ and CD8+) and NK cells [9,13].

Recent *in vitro* experiments revealed that AQP4-IgG is able to cause decreased expression of aquaporin-4 on the astrocyte surface, but also NK cells degranulation and astrocyte death by antibody-dependent cellular cytotoxicity (ADCC) as well as complement-dependent granulocyte infiltration to the CNS through the impaired BBB [7,33].

Selective loss of astrocytic markers such as glial fibrillary acid protein (GFAP) and S-100β protein occurs in NMO lesions suggesting astrocyte damage [16]. AQP4 and GFAP loss is connected with a humoral response against AQP4, which leads to astrocyte death [9,13,16]. AQP4-negative and GFAP-positive astrocytes are present in NMO lesions, which might suggest that loss of AQP4 precedes astrocytic death. When both AQP4 and GFAP are lost, but myelin is preserved, one may expect that astrocyte death precedes myelin destruction [9]. Several studies revealed that there is a rise in GFAP levels in the CSF of NMO patients during disease exacerbations [16,34,35]. CSF-GFAP levels significantly increase during relapses and rapidly decrease after HIMP returning to a nearly normal level [34,35]. Besides the rise in GFAP, myelin basic protein (MBP) is also elevated in the CSF during a relapse. MBP levels decrease after HIMP but still remain high [34]. This variability of the CSF-GFAP and CSF-MBP levels suggests that astrocyte damage is accompanied by myelin destruction which is ongoing even after relapse treatment [34]. Additionally, levels of GFAP variability is accompanied by the increase of S-100β in the CSF of NMO patients during exacerbation and its decrease under HIMP therapy [35]. Moreover, CSF-GFAP and CSF-S-100β strongly correlate with clinical severity measured by EDSS scale and the length of spinal cord lesions visualized by MRI [35]. Importantly, both astrocyte markers, particularly GFAP, are expressed at significantly higher levels than in MS (GFAP even several thousand times higher), acute disseminated encephalomyelitis (ADEM), spinal cord infarction or other neurological diseases [35]. This suggests that CSF-GFAP and CSF-S-100β might be useful biomarkers of astrocytic damage in NMO [34].

Several studies provided evidence that AQP4-IgGs cause complement-depended cytotoxicity (CDC) leading to NMO lesions development [9,20]. Since AQP4-IgG is an IgG1 isotype antibody, it is able to activate the classical complement cascade [9,31,36]. It is reported that during the disease attack total hemolytic complement activity (CH50) is increased in sera of NMO patients who are AQP4-IgG seropositive and have extensive CNS lesions in comparison with those who are AQP4-IgG seronegative or with MS patients [31]. Activation of C3 convertase promotes complement components and production of several proinflammatory cytokines and secretion from astrocytes [31]. Secretion of C3a and C5a factors leads to increased vascular permeability. Component C5a additionally provides a chemotactic gradient resulting in the recruitment of inflammatory cells. Phagocytosis is facilitated by membrane binding of C3b. This sequence of events leads to intense granulocytic infiltration. Complement-mediated injury and cellular infiltration results in dysfunction of astrocytes, and secondarily, astrocyte-dependent cells, such as oligodendrocytes and neurons [31]. Moreover, an association was observed between NMO deterioration and changes in serum concentrations of C4d component [37,38] and CSF levels of C5a [37,39]. Additionally, research showed that increased levels of C3a in sera from NMO patients are associated with disease activity, neurological disability measured by EDSS and AQP4-IgG titers [37]. Interestingly, antibodies against complement C1q (anti-C1q) can be identified in sera of NMO patients and their levels are higher in comparison with MS patients, as was the case with C3a levels, but they do not correlate with disease activity [37]. These observations, combined with immunopathological studies, support the hypothesis of complement involvement in NMO pathogenesis.

Research revealed that in the case of the absence of complement, ADCC can also cause NMO-like lesions, thus it is potentially involved in NMO pathogenesis [20]. This hypothesis is supported by studies in mice, in which injection of AQP4-IgGs and NK cells into mouse brain led to astrocyte injury, marked by AQP4 and GFAP loss [20]. Such AQP4-IgG mediated ADCC leads to minimal myelin loss and inflammation in comparison with lesions produced by AQP4-IgG in a complement-dependent manner [20]. Moreover, NK cells intensify NMO lesions produced by AQP4-IgG and complement, and cause significant myelin loss in *ex vivo* spinal cord slices [20]. Not only can NK cells cause astrocyte damage in the CNS in the absence of complement, but they can also escalate NMO lesions development when complement is present [20]. Besides NK cells, several other leukocyte types can take part in ADCC. AQP4-IgG is predominantly of the IgG1 subtype, so it can bind cells expressing Fc receptors for immunoglobulin G, including macrophages and granulocytes (neutrophils and eosinophils) which are abundantly present in NMO lesions [20]. Nevertheless, the significance of ADCC in NMO is not well defined [20].

Furthermore, research suggests that AQP4-IgG binding to astrocytic AQP4 causes downregulation of excitatory amino acid transporter 2 (EAAT2) and impairs glutamate homeostasis [7,32]. EAAT2 is expressed in astrocytes and it is responsible for over 90% of glutamate uptake in the CNS. It is also essential for glutamate clearance from excitatory synapses [32]. It is reported that EAAT2 and AQP4 are present in astrocytic membranes as a macromolecular complex. AQP4-IgGs cause downregulation of both, EAAT2 and AQP4, from the surface of astrocytes. Because of the fact that astrocytes are relatively tolerant of increased glutamate concentrations, impairment in glutamate homeostasis has especially excitotoxic potential for neurons and oligodendrocytes. A local rise in extracellular glutamate levels can lead to injury or even death of oligodendrocytes expressing calcium-permeable glutamate receptors. Importantly, oligodendrocytes in the spinal cord and in the optic nerves, which are major sites of demyelination in NMO, are particularly sensitive to glutamate concentration changes [32]. Therefore, the role of EAAT2 in NMO pathogenesis could offer new directions in NMO treatment strategies [32].

Finally, several animal models for NMO have been reported [7]. Some of them revealed that administration of AQP4-IgGs can induce NMO-like lesions in Lewis rats with T-cell-mediated experimental autoimmune encephalomyelitis [7,16,22,40]. Another model showed that injection of NMO-IgG together with human complement produces NMO-like lesions in mice without pre-existing CNS inflammation [7,36]. Several animal models provide evidence for AQP4-IgG pathogenicity in NMO and can be very useful for further research in immunopathogenesis of NMO [7].

#### 3.3.2. Synthesis of AQP4-IgG—Is It Only Peripheral or Also Intrathecal?

Several studies suggest that in NMO AQP4-IgGs are not synthesized intrathecally, but are rather formed peripherally and subsequently enter the CNS through a disrupted BBB [9,41].

It is reported that CSF AQP4-IgGs are present in 68% of AQP4-IgG seropostive NMOsd patients, but are absent in AQP4-IgG seronegative patients and in the control group [42]. There are characteristics associated with CSF AQP4-IgG seropositivity, including disease attack within 30 days before the lumbar puncture, high AQP4-IgG serum titers (>1:250) and dysfunction of the BBB [42]. Moreover, AQP4-IgG is identified more frequently during disease deterioration [42].

Several studies found that AQP4-IgGs are more than 500 times more concentrated in serum than in the CSF [29] which leads to the conclusion that AQP4-IgGs are formed peripherally and enter the CNS afterwards [9,42]. Jarius *et al.* [42] reported one patient (1 out of 23 evaluated in the study) with intrathecal AQP4-IgG production and pointed to the lack of quantitative evidence for intrathecal synthesis of this autoantibody in the majority of NMOsd cases [42]. Nevertheless, Bennett *et al.* [22] found that CD138+ plasma cells present in the CSF in early NMO cases are able to synthesize AQP4-IgGs and might be the primary essential source of intrathecal AQP4-IgG production [22]. Consequently, AQP4-IgGs might be synthesized intrathecally at the onset of the disease and directly lead to lesion formation in the CNS [22].

These studies suggest that intrathecal synthesis of AQP4-IgG can occur in NMOsd patients, however, its significance in NMO pathogenesis requires further research [22,42].

#### 3.3.3. How Does AQP4-IgG Enter the Central Nervous System (CNS)?

Interestingly, AQP4-IgG might be present in serum several years before the disease onset [9]. It is supposed that an additional factor that causes increased BBB permeability plays a role here. It is speculated that such factor might be infection, since cases of NMO preceded by viral illnesses or vaccinations were reported [1,43]. However, it still remains unclear whether such event could trigger NMO-associated autoimmunity [43]. Acute viral infection could also trigger autoimmunity via molecular mimicry mechanism [43].

On the other hand, it has been reported that severe BBB damage is not necessary for serum AQP4-IgG to gain access to the CNS [42]. There are areas of the barrier where AQP4-IgG has good access from the microvasculature to astrocytic endfeet, including fenestrations in the pia cell layer at the level of arterioles as well as BBB absence at the level of capillaries, venules and veins [42,44]. This suggests that AQP4-IgG can enter the CNS without BBB disruption via regions of its physiologically increased permeability or by extracellular pathways [42,45]. Moreover, AQP4-IgG itself might lead to damage of BBB, since prolonged exposure to these antibodies in the fenestrated perivascular and subpial spaces could cause local inflammation or AQP4 internalisation resulting in initation of autoimmune response and BBB injury [42,45].

The question why NMO lesions occur specifically in the optic nerves and in the spinal cord remains unanswered. It has been hypothesized that the BBB lacks its classical characteristics or is not fully developed in the prelaminar portion of the optic nerves and in the root entry zones in the spinal cord. Therefore, in these regions of nonspecific permeability circulating AQP4-IgG could have better access to the affected structures than in the other parts of the CNS [9,46,47].

#### 3.3.4. How AQP4-IgG Immune Response Leads to Demyelination?

Research revealed that in NMO, in contrast to MS, demyelination is a process secondary to astrocytic impairment [20,48]. In NMO first AQP4-IgGs taking part in complement-dependent cytotoxicity or antibody-dependent cell-mediated cytotoxicity cause astrocyte damage and then injure oligodendrocytes, which leads to demyelination [48]. This hypothesis is supported by the immunopathological study performed by Misu *et al.* which showed that NMO lesions are marked by the loss of AQP4 and GFAP from the early lesion stage formation, while MBP is relatively preserved [48]. Nevertheless, the mechanism of oligodendrocyte damage is not well-defined [20]. The possible explanations include the following: oligodendrocyte injury occurs in a bystander effect during inflammatory process induced by AQP4-IgG, or it is a result of activated complement components toxicity, or a specific impaired balance between astrocytes and oligodendrocytes [20]. Moreover, demyelination in NMO might be associated with infiltration of inflammatory cells, particularly in the case of complement-dependent cytotoxicity (CDC), as in ADCC, myelin loss is minimal [20].

### 3.4. Detection of AQP4-IgG—Comparison of Sensitivity and Specificity of Different Assays

There are several assays for detection of AQP4-IgG in serum. The principal diagnostic methods are: indirect immunofluorescence assay (IIF), cell-based assay (CBA), radioimmunoprecipitation assay (RIPA), fluoroimmunoprecipitation assay (FIPA) and enzyme-linked immunosorbent assay (ELISA) [25]. All of the above are useful in identifying seropositive patients, however, their sensitivities and specificities differ [25].

Indirect immunofluorescence was the original method used to identify AQP4-IgG as a specific antibody marker for NMO [5,6,25]. This assay has the estimated 54%–73% sensitivity and 91%–100% specificity [5,25,49]. Reduced sensitivity may result from the fact that the mouse tissue used in this method differs from the human one in four extracellular amino acids [25]. On the other hand, the use of tissue substrate allows binding of antibodies to both intracellular and extracellular regions of AQP4, which allows its use as an initial screen to detect AQP4-IgG [25].

**Figure 4:**
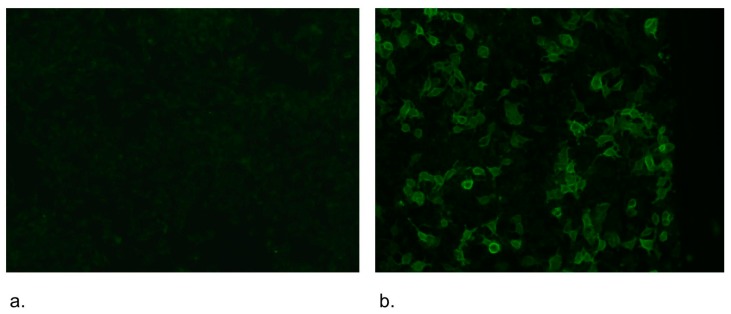
Immunofluorescence staining for aquaporin-4 in a cell-based assay: (**a**) negative stain; (**b**) positive stain. Images from the collection of Division of Neurochemistry and Neuropathology, Department of Neurology, Poznan University of Medical Sciences.

A cell-based assay (CBA) was described for the first time as a proof of AQP-4 detection as a target antigen for NMO-IgG [6,25]. Subsequently, this method was adapted for routine use [25,29,49] (see Figure 4). This CBA has an estimated 91% sensitivity and 100% specificity [25,29].

Radioimmunoprecipitation assay was developed to obtain a test that could be used for everyday practice [25,50]. However, its sensitivity is relatively low (57%) with 98% specificity [25,50].

Fluoroimmunopreciptiation assay is believed to have 76% sensitivity and 100% specificity [25,49]. The advantage of FIPA is that it is highly quantitative and suitable for several estimations [25,49].

Enzyme-linked immunosorbent assay was established to detect anti-aquaporin 4 antibodies [25,51]. The results of 71% sensitivity and 98% specificity are comparable with IIF [25,51].

Waters and Vincent [25] compared the principal methods used for AQP4-IgG detection based on literature and the relapsing NMO cohort [25]. They concluded that IIF, CBA and FIPA have similarly high sensitivity (71%, 91% and 76%) and 100% specificity, except for IIF (94%–100%) [25]. According to their study, RIPA and ELISA were the least sensitive assays (57% and 71%) [25]. IIF is used as a routine initial test that allows AQP4-IgG detection [25]. Nevertheless, CBA seems to be the most sensitive and specific assay [25]. On the other hand, FIPA assay might be a high-throughput test for identifying positive sera and for serial estimations of AQP4-IgG levels [25].

Waters *et al.* [52] performed a multicenter comparison of AQP4-IgG assays using sera from patients with NMO, NMOsd, relapsing-remitting MS, various autoimmune diseases and healthy subjects [52]. The study revealed that the most sensitive assays were quantitative flow cytometry (FACS, fluorescence-activated cell sorting 77%) and CBA (73%). The next most sensitive was ELISA (60%). Importantly, lowering the cutoff value from 5.0 to 1.6 U/mL resulted in the increase of ELISA sensitivity from 60% to 70%, but as it can be expected, it reduces its specificity. FIPA and tissue-based IIF turned out to be the least useful for detecting AQP4-IgG because of low sensitivities (48%–53%). Moreover, CBA and ELISA commercial assays were 100% specific and, respectively, 68% and 60% sensitive for AQP4-IgG. The sensitivity of the commercial CBA and ELISA was 72% when used in combination. In spite of its low sensitivity, FIPA turned out to be appropriate for serial determinations and monitoring patients longitudinally [52].

According to IPND, it is recommended that cell based assays (microscopy or flow cytometry) should be used for AQP4-IgG detection because they have the highest sensitivity and specificity [8]. Indirect immunofluorescence assays and ELISA can also be used, but their results should be interpreted carefully, particularly in case of patients with low-titer positive ELISA result and clinical “red flags” suggesting pathologies other than NMO [8].

When it comes to comparing various assays, there are several problems that should be taken into account. It is essential to know whether a blood sample was taken during relapse or remission, whether the patient has a monophasic or recurrent disease course, and whether the patient has been treated with immunotherapy [25].

It is worth noticing that providing OAPs as a target for antibodies could improve sensitivity of CBA and FIPA [25,53]. For instance, AQP4 M23 transfected Chinese hamster ovary (CHO) cells can provide OAPs. By contrast, the M1 and M23 isoforms of AQP4 can be expressed in human embryonic kidney (HEK) cells, but there is no evidence of arrays forming in these cells [25,53].

### 3.5. Serum Levels of AQP4-IgG—How Do They Change with Disease Activity and during Treatment?

According to several studies, AQP4-IgG titers in serum have clinical and immunological implications [29]. AQP4-IgG levels change depending on disease activity and treatment status. Moreover, several studies suggest that relapse severity may depend on the degree of complement activation initiated by AQP4-IgG [7,29,30,54].

Antibody titers are higher in patients with permanent complete blindness in at least one eye, as well as longitudinally extensive myelitis encompassing at least three vertebral segments, and extensive or large brain lesions visualized on MRI [29]. Moreover, there is a positive correlation between serum AQP4-IgG titers and the length of spinal cord lesions on MRI at the nadir of clinical deterioration [29]. Some patients with short (one to two vertebral segments) spinal cord lesions are also seropositive for AQP4-IgG, but antibody titers in their sera are low and they present other clinical and neuroradiological characteristics of NMO (ON and myelitis, symmetric diencephalic or periaqueductal lesions, intractable hiccup and nausea) [29].

The presence of AQP4-IgG influences the results of multimodality-evoked potentials [55]. A lack of the P100 component on visual-evoked potentials was observed in higher percentage (over 64%) of AQP4-IgG seropositive patients [55]. However, anti-AQP4 immune response did not influence responses and central sensory conduction times in median and posterior tibial nerve somatosensory-evoked potentials (SEPs) [55].

Research suggests that AQP4-IgG titers in NMO/NMOsd are related to disease activity and the risk of relapse [29,30]. AQP4-IgG could be detected in patients’ sera during remission, as well as during relapse, but antibody titers are significantly higher during disease deterioration [30]. In patients with coexisting myasthenia gravis and thyroiditis, the rise in AQP4-IgG levels during disease attack is not connected with any increase in other autoimmune antibodies, for example, to thyroid peroxidase, to thyroglobulin or to acetylcholine receptor [30]. It is reported that AQP4-IgG seropositive patients tend to relapse when antibody titers are high [29] and serum AQP4-IgG levels increase rapidly (~20% per week) and significantly (up to ~290%) shortly before NMO relapse. However, there is no general threshold value for triggering clinical relapse [30].

Interestingly, there are NMO/NMOsd patients who experience a disease attack despite low AQP4-IgG titers and in a few patients treated with immunosuppression high titers are not parallel with clinical deterioration [30]. This does not contradict the pathogenicity of AQP4-IgG, but suggests that other factors might be necessary for tissue damage and clinical signs of the disease, besides the presence of AQP4-IgG [30].

AQP4-IgG titers in sera of NMO patients significantly decrease after treatment with high-dose intravenous methylprednisolone and remain low under an immunosuppressive therapy with oral prednisolone and azathioprine [29,30], as well as cyclophosphamide and rituximab [30]. Therapy with rituximab causes a decrease in AQP4-IgG levels accompanied by decline in CD19 cell number. Nevertheless, AQP4-IgG remains detectable in spite of CD19 cell counts being below the detection limit. In patients treated with rituximab, relapses are preceded by a reoccurrence of CD19 cells. Importantly, even their low numbers can be sufficient to induce rise in AQP4-IgG titers and to initiate the attack [30]. Based on the above, one may conclude that immunosuppressive therapies keep antibody titers low and help to reduce relapse rates [29,30].

According to the IPND, conversion from seronegative to seropositive status is possible and, therefore, retesting should be considered, particularly before plasma exchange or immunosuppressive therapies as well as in sero-negative patients who experience a relapse [8].

Several studies showed that AQP4-IgG is not only a specific biomarker for NMO and a major element of its pathogenicity, but it might also be used as a marker of disease activity [30].

### 3.6. Epidemiological and Clinical Differences between AQP4-IgG Seronegative and AQP4-IgG Seropositive NMO

Research revealed that AQP4-IgG seropositive patients have different clinical and epidemiological features than AQP4-IgG seronegative patients [9,41]. Characteristics associated with seropositivity include female sex, coexisting autoimmunity, severity of clinical attacks and higher total spinal cord lesion load [9,41]. On the other hand, seronegative group commonly presents bilateral optic neuritis at disease onset. Simultaneous optic neuritis and myelitis and shorter time to the diagnosis of NMO after the first relapse are also common among seronegative patients [41]. Some researchers found that relapse frequency tends to be lower among seronegative patients [7,29], however, others reported there are no significant differences between these two groups [41].

No differences have been reported between seropositive and seronegative patients with regards to: age at onset, time to relapse, annualized relapse rate, relapse outcome, annualized EDSS increase, mortality rate, brain lesions visualized on MRI, CSF findings, frequency of preceding infections and history of cancer [41]. It is worth pointing out that patients with monophasic course of NMO are more frequently AQP4-IgG seronegative [7,29,41]. Monophasic course has several other characteristic features, including: a more significant residual deficit after relapses, but better long-term outcome, rare occurrence of respiratory failure and higher five-year survival rate [1]. Reports showed that patients with the relapsing course of the disease have similar clinical characteristics and disease activity regardless of AQP4-IgG status [7,29].

### 3.7. AQP4-IgG Predictive Role

It has been shown that patients with a first event of longitudinally extensive idiopathic acute transverse myelitis, that are seropositive for AQP4-IgG, are at high risk of developing NMO [3,7]. Weinshenker *et al.* [56] reported that 56% of AQP4-IgG seropositive patients with a first-ever LETM experienced a second event within a year [56]. According to this study, AQP4-IgG seronegative patients with LETM neither experience another attack of LETM nor did they develop optic neuritis [56].

### 3.8. What Causes AQP4-IgG Seronegative NMO?

There are several hypotheses to explain the fact that 10%–25% of patients with clinical signs of NMO are seronegative for AQP4-IgG [2]. Firstly, it might be a result of inadequate sensitivity of the currently used assays [2,9]. Therefore, great efforts should be made to improve the sensitivity of assays; Secondly, it could be the matter of diagnostic criteria [2]; Finally, some AQP4-IgG seronegative patients with clinical signs of NMO might have antibodies against others antigens in the astrocytes, similarly as in myasthenia gravis, where patients seronegative for anti-acetylocholine receptor antibodies produce anti-MuSK or anti-titin antibodies [9]. Antibodies against myelin oligodendrocyte glycoprotein (MOG-IgG) have been detected in some patients with AQP4-IgG seronegative NMO as well as with recurrent optic neuritis, longitudinally extensive transverse myelitis, MS and SLE [9].

Interestingly, seropositivity for AQP4-IgG differs depending on geographical localisation and ethnicity. It is reported that 56%–73% of Caucasians, 33.3% of Caribbean, 47% of Italian, 63%–90% of Japanese and 70%–76.9% of Chinese patients with NMO/NMOsd are seropositive for AQP4-IgG [57].

To sum up, AQP4-IgG seronegative NMO is probably a heterogeneous group [9].

## 4. Autoantibodies against Aquaporin-1 (AQP1-Ab)

### 4.1. AQP1-Ab in NMOsd Patients

Recent research revealed that antibodies against aquaporin-1 (AQP1-Abs) might be detected in some patients with NMO or NMOsd [11,58].

Tzartos *et al.* [11] found that AQP1-Ab and AQP4-IgG can both be detected in sera of patients with suspected NMOsd. The double-seropositive patients accounted for 24% of AQP1-IgG seropositives. Moreover, it turned out that AQP1-Abs are more frequent than AQP4-IgGs. Importantly, AQP1-Abs were absent from sera of the healthy individuals and those with neurological autoimmune non-demyelinating disorders (e.g., myasthenia gravis) [11].

Another study by Long *et al.* [58] showed that AQP1-Abs are present in the majority of patients with NMO or at high risk of the disease (in 74.8% AQP4-IgG seropositive patients), but also in some patients with MS and rarely in patients with other neurological disorders [58]. Some patients with NMO or NMOsd and even MS were AQP4-IgG seronegative but AQP1-Ab seropositive or the opposite [58].

Tüzün *et al.* [59] found that the majority of clinically definite NMO patients were AQP1-Ab and AQP4-IgG double-seropositive [59]. Furthermore, they pointed to a disproportion between frequencies of AQP1-Ab seropositivity in the group of patients suspected of NMOsd and fulfilling the revised diagnostic criteria for NMO. It might be explained by the fact that AQP1-Abs are more prevalent in demyelinating diseases that do not fulfill definite criteria for NMO [59].

Research revealed that AQP1-antibodies bind to both the extracellular and cytoplasmic domain of AQP1. Nevertheless, the majority of AQP1-Abs bind to extracellular epitopes [11].

### 4.2. AQP1 Expression in the CNS

Aquaporin-1 is a member of the large AQP family which includes 13 types of AQPs, each with a tetrameric structure, located in the cell membrane and serving as a water channel [11,58]. AQP1 is highly expressed in human astrocytes [11,58], especially in the areas where NMO lesions typically develop [11]. AQP1 is also abundantly expressed in microvascular endothelium, although its function remains unclear [58]. Moreover, in some neurological disorders, *i.e.*, MS, there is increased expression of AQP1 and AQP4 in the brain, probably due to the need to maintain water homeostasis [11]. It has also been reported that AQP1 expression on the astrocyte surface is reduced in some NMO lesions. In addition, AQP1 is present in astrocytic intracellular granules together with AQP4 [11].

### 4.3. AQP1-Ab Mediated Immune Response and Its Possible Pathogenic Role in NMO

At the moment it is unclear whether AQP1-Abs are involved in NMO immunopathogenesis. Nevertheless, there are several findings supporting such a hypothesis [11,58]. AQP1 is highly expressed in human CNS astrocytes and is selectively lost around NMO lesions [11,58]. The high frequency of double-seropositive patients and a correlation between higher antibody titer for either AQP and a greater chance of being double-seropositive might suggest that a related mechanism activating the immune system exists. Finally, AQP1-Abs belong to complement-activating IgG1 subclass, and the majority of them bind to extracellular domain of AQP1 [11]. High expression of AQP1 in microvascular endothelium suggests that AQP1-Ab might lead to BBB disruption and astrocyte damage [58].

### 4.4. AQP1-Ab Assays

Classic assays for NMO-IgG do not detect AQP1-Abs, so different methods are necessary to identify them in sera of patients with suspected NMO or NMOsd [11].

Tzartos *et al.* [11] developed and used radioimmunoprecipitation assay (RIPA) as an initial screening assay and subsequently confirmed the specificity of AQP1-Ab in sera of patients by several methods, including ELISA and Western blotting [11]. Generally, RIPA is more sensitive and specific than ELISA. Since ELISA is preferable in routine diagnostic setting, a simple and sensitive ELISA with intact affinity-pure AQP1 was developed. It turned out that AQP1-ELISA results were concordant with RIPA results in most of the cases, which makes it potentially useful in the near future [11].

Long *et al*. [58] developed cell-based assay (CBA) to detect AQP1-Abs. Test sensitivity was 74.5% in AQP4-IgG seropositive patients and test specificity was 79.6% compared to multiple sclerosis (MS) patients and controls [58].

### 4.5. AQP1-Ab Specificity

Tzartos *et al.* [11] reported that AQP1-Abs have high sensitivity and specificity for NMO [11]. However, Long *et al.* [58] suggested that the specificity was lower than in previous studies and evaluated the diagnostic value of AQP1-Ab as lower than AQP4-IgG in NMO. They did not find any better diagnostic value when both AQP1-IgG and AQP4-IgG were evaluated [58].

### 4.6. Clinical Similarities and Differences between AQP1-Ab Seropositive and AQP4-IgG Seropositive NMO Patients

The low female to male ratio of patients with AQP1-Abs (1.9:1) is equal to that for patients with AQP4-IgGs but lower than for AQP4-IgG seropositive patients (4:1) [11].

The study by Tzartos *et al.* [11] of AQP1-Ab seropositive but AQP4-IgG seronegative patients with suspected NMOsd revealed that almost all of the patients had spinal cord lesions (19 out of 22). The vast majority of the patients (16) had LETM (five of them also ON), one had only transverse myelitis, and two were diagnosed with MS and had mainly significant spinal cord lesions. AQP1-Abs in patients with spinal cord lesions bound predominantly to the extracellular domain of AQP1. Another three out of 22 AQP1-Ab seropositive patients had MS, but almost none of AQP1-Abs in this group were able to bind to extracellular epitopes and bound to cytoplasmic epitopes instead. Moreover, three out of 22 patients were also diagnosed with a neoplasm (nephroma, non-Hodkin lymphoma or mammary cancer), which lead to the conclusion that AQP1-Ab might be considered a paraneoplastic factor like AQP4-IgG [11]. Importantly, AQP1-Ab seropositive NMOsd patients had similar clinical characteristics to AQP4-IgG seronegative ones [11].

Tüzün *et al.* [59] emphasized that all AQP1-Ab and AQP4-IgG double seropositive patients in their study had optic neuritis during the first attack of the disease. This points to the conclusion that AQP1-rich optic nerves could be the initiator of the autoimmune response. The study also showed that AQP4-IgG seropositives had higher EDSS scores than double seropositives and seronegative patients. Moreover, double seropositive and AQP4-IgG seropositive patients had a higher number of relapses than the seronegatives [59].

### 4.7. Is the AQP1-Ab a New Potential Biomarker for NMO?

AQP1-Abs might potentially be a novel biomarker for AQP4-IgG seronegative NMOsd because of their presence in sera of some patients with demyelination in the CNS, abundant expression of AQP1 in astrocytes and similarities with AQP4-seronegative NMO [11].

## 5. Antibodies against Myelin Oligodendrocyte Glycoprotein (MOG-IgG)

### 5.1. MOG-IgG in NMOsd Patients

It is reported that antibodies against myelin oligodendrocyte glycoprotein (MOG-IgGs) are present in sera of some NMO patients that are seronegative for anti-aquaporin-4 antibodies (AQP4-IgGs) [12,60].

### 5.2. MOG-IgG Expression in the CNS

Myelin oligodendrocyte glycoprotein (MOG) is present on the outer surface of myelin sheaths in the CNS and accounts for about 0.05% of total myelin protein [12]. MOG-IgGs bind to extracellular domains of MOGs, which might result in crosslinking and internalization of MOGs as well as reversible retraction of oligodendrocyte processes [12].

### 5.3. How Does MOG-IgG Cause Lesions in the CNS and Is It Potentially Pathogenic in Vivo?

It remains unclear whether MOG-IgGs play a pathogenic role in NMO patients *in vivo*. According to studies in mice, there is a possibility that MOG-IgGs might cause lesions in the CNS [12,60].

MOG-IgGs cause temporary damage of myelin and axons and alter axonal proteins’ expression after injection into the mouse brain. These changes are complement-independent and reversible within two weeks. Importantly, MOG-IgGs do not produce inflammatory cell infiltration, axonal loss, neuronal or astrocyte death [12].

MOG-IgGs cause altered myelin basic protein (MBP) and reduced axonal Caspr and AnkG expression which are essential for the nodes of Ranvier integrity and normal potential firing. Changes in MBP and axonal protein expression might lead to lesion formation in the CNS [12].

Currently, it remains unknown how MOG-IgGs induce myelin damage *in vivo*. Research suggests that myelin impairment is a direct effect of MOG-IgG binding and activation of the complement cascade is unnecessary. MOG-IgG binding might result in MOG conformational changes and internalization, which in turn leads to changes in myelin structure [12]. There are also hypotheses explaining minimal, or the lack of, complement activation, including MOGs inability to aggregate because of its low abundance after MOG-IgGs binding as well as MOGs internalization due to MOG-IgGs binding and thus prohibiting C1q activation [12]. Nevertheless, it was also reported that MOG-IgGs mainly belong to the IgG1 subtype and are able to mediate complement-dependent cytotoxicity at high-titer levels [60,61].

### 5.4. MOG-IgG Specificity

According to current studies, MOG-IgG might be present in NMO, but also in other neurological disorders, including MS and ADEM, and even in some healthy individuals [12,60]. The question whether MOG-IgG from NMO and non-NMO patients could cause the same CNS changes remains unanswered. The assays for MOG-IgG should be developed and standardized to determine which subpopulation of MOG-IgG could lead to the CNS destruction and in which diseases [12].

### 5.5. Clinical Characteristics of MOG-IgG Seropositive NMOsd Patients

Recent research revealed NMO patients with MOG-IgG have several demographic, clinical and radiological characteristics in comparison with those who are AQP4-IgG seropositive or seronegative [61,62].

Sato *et al.* [61] found that MOG-IgG seropositive patients are more frequently male [61]. The median age at onset is similar to the one of AQP4-IgG seropositives (respectively 37.5 and 37) and higher than in the seronegatives (32.5) [61].

Patients with MOG-IgG tend to have a single or a lower number of disease attacks. Bilateral, simultaneous optic neuritis attacks are more common in patients with MOG-IgG than in those with AQP4-IgG or in seronegative ones [61]. Brainstem symptoms (nausea, vomiting and hiccups) and painful tonic spasms were significantly less frequent in MOG-IgG seropositives [61]. Moreover, they usually demonstrate better recovery from the attack [61]. MOG-IgG seropositive patients are marked by a better outcome in comparison with AQP4-IgG seropositive patients regardless of the course of the disease [12,61]. However, there are some MOG-IgG seropositive patients who experience severe disability after disease relapse and do not recover well [61].

Brain lesions visualized on MRI are more common in AQP4-IgG seropostitive and seronegative patients (approximately 56%) than in MOG-IgG seropositive patients (37.5%) [61]. Spinal cord lesions on MRI are also significantly less frequent in MOG-IgG seropositives (37.5%) than in AQP4-IgG seropositives (92.1%) or seronegatives (71.7%) [61]. Additionally, lesions in patients with MOG-IgG are usually distributed in the lower parts of the spinal cord, in the thoracolumbar region, as opposed to patients with AQP4-IgG or seronegatives who have more lesions in the cervicothoracic region [61].

Interestingly, antinuclear antibodies (ANA) are less frequent in MOG-IgG seropositive patients than in those who are AQP4-IgG seropositive or seronegative [61].

A study by Ramanathan *et al.* [62] revealed that there is a strong relationship between MOG-IgG seropositivity and bilateral and/or recurrent optic neuritis in AQP4-IgG seronegative patients (sensitivity 69% and specificity 99%) [62]. Most of the MOG-IgG-seropositive patients in this study were females (in contrast to the study by Sato *et al.* 2014 [61]) with a relapsing course of the disease, a rapid response to steroid therapy, relapses after steroid cessation and good follow-up visual acuity [62].

### 5.6. MOG-IgG Seropositive Patients Treatment

Current research findings suggest that if MOG-IgG is involved in NMO pathogenesis, treatment with steroids or plasma exchange should be effective in MOG-IgG seropositive NMO patients. Several new potential therapies for AQP4-IgG seropositive NMO patients, like sivelestat (inhibiting neutrophils) or eculizumab (inhibiting complement), are probably less effective in NMO patients with MOG-IgG [12].

### 5.7. MOG-IgG—Perspectives

MOG-IgG is a new potential biomarker of NMO/NMOsd [57]. Moreover, the latest research suggests that MOG-IgG might be involved in immunopathogenesis of neuromyelitis optica. Nevertheless, further *in vivo* studies are necessary to fully elucidate the role of MOG-IgG in disease diagnosis and in producing NMO lesions [12,57].

## 6. Other Potential Biomarkers in NMO

There are several factors potentially useful for NMO/NMOsd diagnosis, prediction of disease attacks and its course as well as evaluation of treatment efficacy [57].

Astrocyte markers, such as previously mentioned GFAP and S-100β protein, might be useful biomarkers of disease activity and astrocytic damage in NMO patients [34,57]. Several studies revealed that CSF-GFAP and CSF-S-100β levels change with disease activity [27,62,63] and strongly correlate with clinical severity [35].

Apart from AQP4-IgG, AQP1-Ab and MOG-IgG other antibodies might be detected in NMO/NMOsd patients, e.g., antibodies against *N*-methyl-d-aspartate-type (NMDA-type) glutamate receptor, against collapsin response mediator-protein 5 (CV2/CRMP5) and against glycine receptor [57].

It has been reported that several cytokines, chemokines and other markers of inflammation are also present in sera or in the CSF of patients with NMO/NMOsd. These are mostly associated with T_H_2 cellular immune response, such as serum interleukin-5 (IL-5) and eotaxin-2 (CCL24), eotaxin-3 (CCL26) in the CSF, B-cell activating factor (BAFF) in serum and in the CSF [57]. Additionally, T_H_17-associated cytokines (*i.e.*, IL-17A and IL-6) and T_H_1-associated interferon-γ are elevated in some patients [57]. Other factors found in sera or in the CSF of some NMO patients are: IL-1 receptor antagonist (IL-1ra), IL-6, IL-8 (CCL8), IL-13, granulocyte colony-stimulating factor (G-CSF), High Mobility Group Box 1 Protein, B lymphocyte chemoattractant (BLC, CXCL13), interferon-gamma-inducible protein-10 (IP-10, CXCL10), and IL-13—responsive chitinase [57]. Since these markers of inflammation are also present in other systemic or inflammatory disorders, their role in NMO needs further research [57].

According to recent research, there are several differences in CSF cytokine/chemokine profiles between NMO and MS patients. The levels of IL-6, IL-8, IL-13, interleukin-1 receptor antagonist (IL-1Ra) and G-CSF are remarkably elevated in the CSF of patients with NMO. On the other hand, IL-9, tumor necrosis factor-α (TNF-α), macrophage inflammatory protein-1-β (MIP-1β), granulocyte macrophage colony-stimulating factor (GM-CSF) and fibroblast growth factor-basic (FGF-basic) are elevated in the CSF of MS patients [64]. Interferon-γ-inducible protein-10 (IP-10) and IL-10 are higher in both NMO and MS patients than in those with other non-inflammatory neurological disorders [64]. Importantly, several studies have pointed to the potential value of CSF IL-6 in diagnosing and monitoring NMO patients [64,65]. IL-6 levels are significantly higher in the CSF of patients with NMO than with MS and other neurological disorders (e.g., peripheral nervous disorders, amyotrophic lateral sclerosis, encephalopathy and degenerative diseases) [65]. The levels of CSF IL-6 correlate strongly with CSF GFAP levels and CSF cell counts as well as are associated with AQP4-IgG titers [64,65]. It has also been reported that IL-6 and GFAP are elevated in the CSF of patients who experienced the first NMO attack and their high sensitivities (76.9% and 84.6% respectively) are similar to those of AQP4-IgG in the serum, which suggests that CSF IL-6 and GFAP might be valuable biomarkers of NMO [66]. Wang *et al.* [67] found that soluble form of IL-6 receptor, which is a valuable IL-6 cofactor, is also increased in the CSF of NMO patients and is strongly associated with clinical disability measured by EDSS [67]. Moreover, it has been found that CSF IL-6 levels are associated with patient clinical condition and can be used to predict recovery after the attack. This hypothesis is further supported by the observation that patients with low CSF IL-6 titers are marked by longer remission periods, and larger clinical improvement measured by EDSS after relapse [68]. To sum up, the above findings point to the conclusion that CSF IL-6 plays a role in NMO immunopathogenesis and is a potential biomarker of diagnosis, disease activity and prognosis [64,65,66,67,68].

Moreover, it has been observed that NMO deterioration correlated with changes in the concentration of several complement components, such as C3a and C4d in serum [37,38] and C5a in the CSF [37,39]. Interestingly, the levels of antibodies against complement C1q (anti-C1q) and C3a are significantly higher in sera of NMO than MS patients [37].

Factors indicating the BBB breakdown might also be used as biomarkers of disease activity in NMO patients [57]. Potential candidates include: matrix metalloproteinase-9 (MMP-9), vascular endothelial growth factor-A (VEGF-A), intercellular adhesion molecule-1 (ICAM-1) and vascular cell adhesion molecule-1 (VCAM-1) [57]. Matrix metalloproteinase-9 is an enzyme causing BBB breakdown due to collagen IV degradation, thus participating in the pathogenesis of several diseases of the CNS, including NMO [69]. It has been reported that MMP-9 levels are remarkably increased in sera of NMO patients as compared with those with MS or healthy individuals and they correlate with CSF/serum albumin ratio as well as the degree of disability measured by EDSS. In addition, MMP-9 serum levels are strongly associated with CSF IL-8 levels, which in turn promote MMP-9 production from neutrophils [69]. According to recent research, VEGF-A might lead to BBB disruption in demyelinating diseases [57]. Shimizu *et al.* [70] showed that antibodies against human brain microvascular endothelial cells (BMECs), which are present in sera of NMO patients, cause BBB breakdown due to increased autocrine VEGF production by BMECs [70]. Another study revealed that soluble ICAM-1 (sICAM-1) and soluble VCAM-1 (sVCAM-1) levels are elevated in the CSF of NMO patients. Additionally, sICAM-1 levels are also increased in NMO patients’ sera. Importantly, sICAM-1 levels in the CSF are strongly associated with CSF cell counts, CSF protein levels and albumin quotient. Moreover, both sICAM-1 and sVCAM-1 CSF levels are correlated with the presence of active, gadolinium-enhanced CNS lesions on MRI [71]. Although AQP4-IgG remains the most important pathological factor in NMO, further longitudinal research in the biomarker field is still needed [57].

## 7. NMO and Other Autoimmune Diseases

### 7.1. NMOsd in the Context of Other Autoimmune Diseases

Several studies showed that there is a strong association between LETM or optic neuritis (ON) and other autoimmune diseases, especially systemic lupus erythematosus (SLE) and Sjögren’s syndrome (SS) [1,2,7,63], but also autoimmune hypothyroidism, pernicious anemia, immune thrombocytopenic purpura, primary sclerosing cholangitis and ulcerative colitis [1,7]. It is estimated that about one third of NMO patients suffers from other autoimmune diseases [1,7]. The frequencies of coexisting systemic autoimmune diseases, as well as the presence of non-organ specific autoantibodies, are similar in NMO and MS. However, it turned out that NMO patients frequently have a family history of autoimmune diseases [2]. One could conclude that NMO is a manifestation of a genetic tendency toward humoral autoimmunity. An alternative theory is that NMO is a complication of a systemic autoimmune disease [63]. If NMO would in fact be a systemic autoimmune disease complication, its onset should occur after the diagnosis of the primary disease. Nevertheless, there have been reports of patients who develop SLE or SS before NMO onset as well as those who manifest NMO before the diagnosis of SLE or SS [63]. NMO typically coexists with thyroid disease (the most common co-existing autoimmune disease), myasthenia gravis or celiac disease [63]. According to several studies, the occurrence of optic neuritis and/or transverse myelitis in AQP4-IgG seropositive patients with systemic autoimmune disease (e.g., SS or SLE) should not be regarded as a vasculitic complication of a systemic disorder, but as the coexistence of these two diseases [2,63].

There are several possible mechanisms explaining co-association between NMO and systemic autoimmune diseases. Firstly, as previously mentioned, it might result from common genetic and/or environmental factors predisposing to autoimmunity. Secondly, systemic autoimmunity might facilitate crucial events in NMO immunopathogenesis, for example, autoantibodies production or other inflammatory mechanisms leading to the BBB disruption. Furthermore, systemic autoimmune diseases might be associated with a common immunopathological mechanism, such as vasculopathy. Finally, AQP4-IgG production might be an epiphenomenon, without direct pathogenic meaning, representing the result of a secondary autoimmune sensitization to autoantigens [63].

The coexistence of NMO with other autoimmune disorders has several clinical implications; most importantly in the differential diagnosis field. If optic neuritis or transverse myelitis occurs in a patient with already diagnosed systemic autoimmune disease, AQP4-IgG assay should be carried out. According to currently accepted standards, if the result of AQP4-IgG is positive, diagnosis of two coexisting disorders, *i.e.*, NMO and systemic autoimmune disease should be made. If the result is negative, careful clinical follow-up is recommended [63].

According to IPND, the presence of SLE, SS or myasthenia gravis in patients with NMOsd is not only typical, but should even be regarded as supportive for NMOsd diagnosis [8].

### 7.2. Autoantibodies in NMOsd Patients

Several studies showed that antinuclear antibodies (ANA) are present in sera of approximately half of NMO patients [2,7]. Non-organ-specific antibodies (especially anti-Ro) are more common in patients with relapsing course of NMO and recurrent transverse myelitis (77%) than in those with a monophasic course of the disease (33%) [2].

The detection of ANA in patients with ON or might suggest that these disorders are vasculitic neurological complications of another systemic autoimmune disease [2]. However, patients with optic neuritis and/or myelitis with non-organ specific antibodies (e.g., ANA) in serum, but without recognized systemic autoimmune disorder, tend to have NMO or NMOsd rather than “lupus myelitis” or “Sjogren-related myelopathy”, regardless of AQP4-IgG serological status [7,63].

### 7.3. NMO in the Context of Myasthenia Gravis and Neoplasms

It is reported that myasthenia gravis is more common in NMO patients than in the general population [7,72]. Moreover, one or more neuronal, glial or skeletal muscle antibodies could be detected in sera of NMO patients, which is more common than in MS or in the healthy individuals. In addition, neuromuscular junction acetylocholine receptor (AChR) antibodies characteristic for myasthenia gravis, can be detected in sera of NMO patients, but are absent in MS and in the healthy controls [72].

It has been speculated that AQP4-IgG in some patients with NMO might reflect a paraneoplastic immune response [73]. Pittock and Lennon [73] reported a correlation between seropositivity for AQP4-IgG and cancer. It turned out that among patients in whom AQP4-IgG was detected incidentally in the course of a paraneoplastic evaluation, 93% had symptoms and signs of NMOsd and 27% had coexisting neoplasm, including breast, lung, thymic and uterine cervical cancer, B-cell lymphoma and monoclonal gammopathy. The clinical signs of NMOsd followed the diagnosis of cancer in most cases, but they might also precede the diagnosis of cancer. Additionally, in this group there were patients (7%) who had cancer and neurological symptoms attributable to CNS metastases [73]. In the group of patients with diagnosed NMOsd, neoplasms were recorded in 5% of cases, including breast cancer, thyroid Hurthle cell carcinoma, carcinoid, pituitary somatotropinoma and B-cell lymphoma [73]. AQP4-IgG is one of the glial-reactive IgG autoantibodies recognized in a paraneoplastic context. Further studies are obviously needed in this context [73].

## 8. Treatment Strategies

### 8.1. Acute NMO Treatment: Methylprednisolone and Plasma Exchange

An intravenous corticosteroid therapy with methylprednisolone (1000 mg daily for five days) is the standard treatment in acute attacks of NMO [2,7,74]. Plasma exchange can be carried out in patients without appropriate response to corticosteroids [2,7,74]. These patients benefit from the course of seven plasma exchanges (1.0 to 1.5 plasma volume per exchange) over a period of two weeks [2,7]. However, in certain situations early treatment with plasma exchange is recommended, especially in NMO patients who experience severe cervical myelitis and because of that they are at risk of respiratory failure [2]. Additionally, plasma exchange is indicated for patients suffering from an acute, severe loss of vision which is resistant to corticosteroid therapy [2].

### 8.2. Maintenance Treatment

Maintenance therapy with immunosuppressant drugs is a commonly used method for reducing frequency of NMO attacks [2]. The first line treatment is usually oral steroid therapy for two to three months, however, in MOG-IgG seropositive patients’ treatment should be prolonged for up to 12 months. Observational studies revealed that the combination of azathioprine (2.5–3.0 mg/kg/day) and oral prednisone (1.0 mg/kg/day) reduced the frequency of relapses [2,7]. The protocol is to establish azathioprine monotherapy by reducing dose of prednisone when azathioprine reaches its complete effect (4–6 months) [7]. Other observational studies suggested that mycophenolate mofetil [7,75], mitoxantrone [2,7,76], intravenous immunoglobulin [2,7,77] and rituximab (the chimeric anti-CD20 monoclonal protein that selectively depletes B cells) [2,7,78] can cause remission of the disease in patients who are refractory to other attempts of treatment [2].

Research revealed that immunomodulatory therapies which are accepted for MS treatment (*i.e.*, beta interferons or glatiramer acetate) are probably ineffective in NMO. Moreover, it has been reported that interferons might even aggravate the disease course [7].

### 8.3. New Directions in the Treatment of NMO

According to recent studies on NMO, there are several new treatments targeting specific components of disease immunopathogenesis [9].

Aquaporumab, which is a non-pathogenic recombinant monoclonal antibody that blocks binding of AQP4-IgG in sera of NMO patients, eliminates complement- and cell-mediated cytotoxicity. It has been shown that aquaporumab is able to prevent the development of NMO lesions in a spinal cord slice model *ex vivo* and in a mouse model *in vivo* [9,79].

Since several studies showed that neutrophils are involved in NMO pathogenesis, especially in the early lesions [9,14], sivelestat, which is a neutrophil protease inhibitor, might be a potential treatment for NMO [9,14]. It has been reported that sivelestat reduces lesions in mice and *ex vivo* due to inhibiting neutrophil entry into the lesion and tissue damage produced by neutrophil elastase [9].

There are also studies on the use of eculizumab in the therapy of NMOsd [9,80]. Eculizumab is a monoclonal immunoglobulin G that inhibits complement. It has been reported that eculizumab caused significant decrease in disease attack frequency as well as stabilisation of disease progression, or even improvement of neurological condition in patients with severe NMOsd. This therapeutic monoclonal IgG is well tolerated. However, therapy with eculizumab is associated with the risk of meningococcal meningitis which can be reduced by administration of meningococcal vaccine [80].

Another promising therapeutic option is tocilizumab [9,81]. Tocilizumab (TCZ) is a humanized anti-interleukin-6 antibody which causes interleukin-6 receptor blockade. Treatment with TCZ resulted in decrease of the annualized relapse rate, reduced EDSS score as well as significant alleviation of neuropathic pain and general fatigue [81].

New treatment strategies warrant further randomized clinical trials before they are introduced into everyday practice [9]. Pharmacoclinical studies are also essential in the context of unravelling NMO immunopathophysiology as a proof of concept.

## 9. Discussion

Recent studies on NMO/NMOsd revealed many novel, interesting, and often surprising facts about this disease and its immunopathogenesis. Although previously believed to be a primarily demyelinating disease and a variant of multiple sclerosis, it turned out to be an autoimmune astrocytopathy, distinct from MS [2,7]. Nevertheless, there are still many controversies and questions that remain unanswered. The primary immunizing event remains unknown [2]; there are several ambiguous aspects of the pathogenic role of AQP4-IgG [2,7,9,16] and other potential factors may play a role in NMO pathogenesis, e.g., AQP1-Ab and MOG-IgG [11,12].

As previously mentioned, several types of immune cells are involved in NMO lesion formation, but their exact role in NMO pathogenesis, which might have important implications for future treatment possibilities, remains unclear [9,13,14,20,21].

Interestingly, the fact that AQP4-IgG might be present in serum for years before the disease onset [9] suggests that some unknown additional factor triggering the inflammatory cascade exists [2]. It is believed that AQP4-Igs are formed peripherally and then enter the CNS [9,42]. However, some studies showed an intrathecal synthesis of AQP4-IgG in NMO patients, but its significance in NMO pathogenesis requires further research [22,42]. There are still several hypotheses on how AQP4-Igs gain access to the CNS. It is not known whether there is another independent factor increasing the BBB permeability [43], or whether AQP4-IgG has independent ability to generate the BBB damage [42,45]. It could also be that AQP4-IgGs enter the CNS via regions of its physiologically increased permeability, or by extracellular pathways without the BBB damage [42,45]. The disruption of BBB and its mechanisms remain an unresolved problem in NMO pathogenesis. The translation of experimental data into the clinical setting is limited by the lack of specific and clinically useful biomarkers of BBB breakdown. Further studies on non-AQP4-Ig-mediated pathomechanisms involved in the increased BBB permeability are required.

What is more, it has been proven that demyelination is secondary to astrocytic impairment [20,48], but the mechanism of oligodendrocyte damage is not well-defined [20]. Another interesting fact is that although AQP4-Igs are believed to lead to astrocyte death through complement-dependent cytotoxicity, research revealed that in the absence of complement antibody-dependent cellular cytotoxicity can also cause NMO-like lesions, however, its significance in NMO pathogenesis needs further research [20]. Finally, absence of AQP4-IgG in some NMO patients suggests that another factor might be involved in NMO immunopathogenesis, e.g., AQP1-Ab and MOG-IgG [11,12].

The results of several studies suggest that AQP4-IgG is not only a specific biomarker for NMO and a major element of its pathogenicity, but it might also be used as a marker of disease activity, a response to treatment and a prognostic factor [3,7,30,56]. Nevertheless, usefulness of serial AQP4-IgG testing with the aim of monitoring disease activity or making a prediction of the disease course needs further longitudinal studies which might result in precise clinical recommendations [57].

Nowadays AQP4-IgG seronegativity in NMO patients is a challenging problem. Anti-aquaporin-1 antibodies are detected in some NMO patients who are seronegative for AQP4-IgG, as well as in patients seropositive for AQP4-IgG. Importantly, these autoantibodies are absent in the healthy individuals, however, opinions about AQP1-Ab specificity and potential diagnostic value differ between studies. AQP1-Ab is a new promising biomarker of this disease and might be involved in NMO immunopathogenesis [11,58]. Another potential biomarker is MOG-IgG which can be present in sera from AQP4-IgG seronegative NMO patients and in other neurological disorders (ADEM, paediatric MS) [57]. It has been speculated that MOG-IgG seropositive subgroup might be the one that in fact corresponds to the original Devic’s description. Importantly, seropositivity for MOG-IgG might have therapeutic implications, as patients with MOG-IgG tend to respond well to steroids and plasma exchange, but are resistant to sivelestat or eculizumab due to minimal complement activation [12].

New treatment strategies targeting specific components of NMO immunopathogenesis created on the basis of detailed and modern knowledge of this disease would be a great step forward [9].

## 10. Conclusions

**Figure 5:**
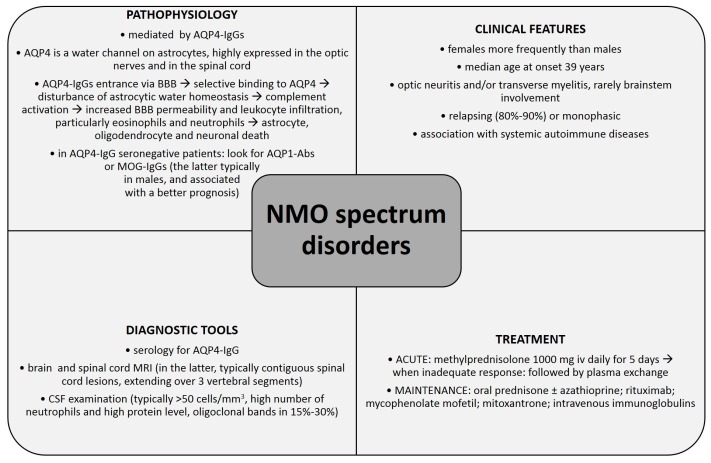
Basic summary of the current knowledge on NMO spectrum disorders. Abbreviations: AQP4—aquaporin 4; BBB—blood-brain barrier; IgG—immunoglobulin G; MRI—magnetic resonance imaging; CSF—cerebrospinal fluid.

Constant efforts should be made to broaden our knowledge of neuromyelitis optica, particularly to understand better its pathogenesis due to its several implications for clinical practice. The summary of the current status of knowledge on NMO is presented in Figure 5. The knowledge about AQP4-IgG and other potential biomarkers of this disease should be developed to facilitate an early and accurate diagnosis and to determine tools for monitoring and predict the disease course. Modern treatment strategies should be created on the basis of detailed knowledge of immunopathogenesis of NMO. An early diagnosis and an effective therapy are essential because NMO is a disease distinct from MS, has a poor prognosis and leads to a neurological disability.

## Abbreviations

AChR: neuromuscular junction acetylocholine receptor
ADCC: antibody-dependent cellular cytotoxicity
ADEM: acute disseminated encephalomyelitis
ANA: antinuclear antibodies
APC: antigen presenting cell
AQP1-Ab: aquaporin-1 antibody
AQP4-IgG: aquaporin-4 immunoglobulin G
BAFF: B-cell activating factor
BBB: blood-brain barrier
BMECs: human brain microvascular endothelial cells
CBA: cell-based assay
CCR3: CC-chemokine receptor-3
CDC: complement-depended cytotoxicity
CH50: total hemolytic complement activity
CNS: central nervous system
CSF: cerebrospinal fluid
DTI: diffusion tensor imaging
EAAT2: excitatory amino acid transporter 2
EDSS: the Expanded Disability Status Scale
ELISA: enzyme-linked immunosorbent assay
FIPA: fluoroimmunoprecipitation assay
FGF-basic: fibroblast growth factor-basic
G-CSF: granulocyte colony-stymulating factor
GFAP: glial fibrillary acid protein
GM-CSF: granulocyte macrophage colony-stimulating factor
HEK: human embryonic kidney
HIMP: high-dose intravenous methylprednisolone therapy
HLAs: human leukocyte antigens
ICAM-1: intercellular adhesion molecule-1
IIF: indirect immunofluorescence assay
IL: interleukin
IL-1Ra: interleukin-1 receptor antagonist
IPND: International Panel for NMO Diagnosis
LETM: longitudinally extensive transverse meylitis
MAC: membrane attack complex
MBP: major basic protein
MG: myasthenia gravis
MIP-1β: macrophage inflammatory protein-1-β
MMP-9: matrix metalloproteinase-9
MOG-IgG: myelin oligodendrocyte glycoprotein immunoglobulin G
MRI: magnetic resonance imaging
Gd (+): gadolinium enhancing
MS: multiple sclerosis
MTR: magnetisation transfer
NE: neutrophil elastase
NMO: neuromyelitis optica
NMO-IgG: neuromyelitis optica immunoglobulin G
NMOsd: neuromyelitis optica spectrum disorder
OAPs: orthogonal arrays of particles
ON: optic neuritis
PB: plasmablasts
PRES: posterior reversible encephalopathy syndrome
RIPA: radioimmunoprecipitation assay
SEPs: posterior tibial nerve somatosensory-evoked potentials
sICAM-1: soluble intercellular adhesion molecule-1SLE: systemic lupus erythematosus
SS: Sjögren’s syndrome
sVCAM-1: soluble vascular cell adhesion molecule-1
TCZ: tocilizumab
TNF-α: tumor necrosis factor-alpha
VCAM-1: vascular cell adhesion molecule-1
VEGF-A: vascular endothelial growth factor-A
